# Factors associated with chemsex in Portugal during the COVID-19 pandemic*

**DOI:** 10.1590/1518-8345.4975.3474

**Published:** 2021-08-30

**Authors:** Jeremias Salomão Chone, Shirley Verônica Melo Almeida Lima, Inês Fronteira, Isabel Amélia Costa Mendes, Ahmed Nabil Shaaban, Maria do Rosário Oliveira Martins, Álvaro Francisco Lopes Sousa

**Affiliations:** 1 Universidade Nova de Lisboa, Instituto de Higiene e Medicina Tropical, Global Health and Tropical Medicine; Lisboa, LS, Portugal.; 2 Universidade Federal do Sergipe, Departamento de Enfermagem, Lagarto, SE, Brazil.; 3 Universidade de São Paulo, Escola de Enfermagem de Ribeirão Preto, PAHO/WHO Collaborating Centre for Nursing Research Development, Ribeirão Preto, SP, Brazil.; 4 Scholarship holder at the Conselho Nacional de Desenvolvimento Científico e Tecnológico (CNPq), Brazil.

**Keywords:** Sexual and Gender Minorities, Coronavirus Infections, Public Health Nursing, Pandemics, Sexual Behavior, Illicit Drugs, Minorias Sexuais e de Gênero, Infecções por Coronavirus, Enfermagem em Saúde Pública, Pandemias, Comportamento Sexual, Drogas Ilícitas, Minorías Sexuales y de Género, Infecciones por Coronavirus, Enfermería en Salud Pública, Pandemias, Conducta Sexual, Drogas Ilícitas

## Abstract

**Objective:**

to investigate the factors associated with the practice of sex under the influence of drugs (chemsex) among Portuguese men who have sex with men during the period of social distancing to prevent the COVID-19.

**Method:**

online survey applied in May 2020 to a sample of 1,301 participants living in Portugal, recruited according to Respondent Driven Sampling and via social media Facebook^®^. Descriptive and bivariate analyses were performed along with logistic regression to calculate adjusted Odds Ratio (ORa).

**Results:**

the prevalence of *chemsex* was 20.2%. The likelihood of practicing *chemsex* increased with group sex (ORa: 28.4, 95%CI 16.93–47.49); unprotected sex (ORa: 7.1 95%CI 4.57–10.99); the use of pre-exposure prophylaxis (PrEP) to prevent COVID-19 (ORa: 4.2, 95%CI 2.71–6.39) and COVID-19 testing (ORa: 1.9, 95%CI 1.15–3.10).

**Conclusion:**

the practice of *chemsex* among men who have sex with men during the COVID-19 pandemic in Portugal was very frequent and may support greater understanding of the role and impact of sexual behavior on the COVID-19 transmission rates and the current pandemic situation in Portugal.

## Abstract (pt)

**Objetivo:**

investigar os fatores associados à prática do sexo sob o efeito de drogas (*chemsex*) entre homens que fazem sexo com homens (HSH) portugueses durante o período de distanciamento social pela COVID-19. **Método**: inquérito *on-line* aplicado em maio de 2020 a uma amostra de 1301 participantes residentes em Portugal recrutados pelo método *Respondent Driven Sampling* na rede social Facebook^®^. Realizaram-se as análises descritiva e bivariada e a regressão logística para o cálculo dos *Odds ratio* ajustado (ORa).

**Resultados:**

a prevalência de *chemsex* foi de 20,2%. A chance de praticar c*hemsex* aumentou com: o sexo grupal (ORa: 28.4, IC95% 16.93 – 47.49); o não uso de preservativo (ORa: 7,1 IC95% 4,57 – 10,99); fazer uso da profilaxia pré-exposição (PrEP) como medida protetiva para a COVID-19 (ORa: 4,2, IC95% 2,71 – 6,39) e realizar teste para a COVID-19 (ORa: 1,9, IC95% 1,15 – 3,10).

**Conclusão:**

a prática de *chemsex* entre homens que fazem sexo com homens, no período da pandemia da COVID-19 em Portugal, foi elevada e pode fornecer subsídios para entender o papel e o impacto que as relações sexuais possuem nas taxas de transmissão e na atual situação pandêmica no país.

## Abstract (es)

**Objetivo:**

investigar los factores asociados a la práctica de sexo bajo el efecto de drogas (*chemsex*), entre hombres que tienen sexo con hombres portugueses, durante período de distanciamiento social por COVID-19. **Método**: encuesta online aplicada en mayo de 2020 a una muestra de 1.301 participantes, residentes en Portugal, reclutados por el método *Respondent Driven Sampling* en la red social Facebook^®^. Se realizó análisis descriptivo, bivariado y regresión logística para cálculo de los *Odds Ratio* ajustado (ORa).

**Resultados:**

la prevalencia de *chemsex* fue de 20,2%. La chance de practicar c*hemsex* aumentó con: el sexo grupal (ORa: 28.4, IC95% 16.93 – 47.49); no usar preservativo (ORa: 7,1 IC95% 4,57 – 10,99); hacer uso de la profilaxis antes de la exposición (PrEP) como medida protectora para la COVID-19 (ORa: 4,2, IC95% 2,71 – 6,39) y realizar el test para COVID-19 (ORa: 1,9, IC95% 1,15 – 3,10). **Conclusión**: la práctica de *chemsex* entre hombres que tienen sexo con hombres, en el período de la pandemia de la COVID-19, en Portugal, fue elevada y puede suministrar informaciones para entender el papel e impacto que relaciones sexuales poseen en las tasas de transmisión y actual situación de la pandemia en el país.

## Introduction

Cases of respiratory infection of unknown origin were first reported in December 2019 in Wuhan, capital of the Hubei province, China and was later named COVID-19 (Coronavirus Disease)^(1)^. In March 2020, the World Health Organization (WHO) considered it a pandemic, and by December 2020, the disease had spread to approximately 218 countries/territories^(2)^. Transmission of the causative virus, the Severe Acute Respiratory Syndrome Coronavirus 2 (SARS-CoV-2), mainly occurs through respiratory droplets and containment and mitigation measures (e.g., social distancing, social isolation and quarantine) and the use of face masks are the primary strategies to prevent infection^(3-4)^.

In some countries, mitigation measures were rigidly and effectively implemented, as was the case of Portugal^(5-6)^. This European country acted early against the SARS-CoV-2 threat. The first cases of COVID-19 were recorded on March 2^nd^, 2020 and 15 days later, the borders with Spain, a country facing one of the worst situations of the pandemic at the time, were closed and the faceto-face teaching was suspended in schools. Days later, the Portuguese government issued a state of emergency and imposed restrictions on people’s circulation, social interaction, and religious services, a total ban on beaches, the closure of non-essential activities and the home office regime was adopted, while new sanitary rules were imposed for the businesses that remained open^(5-7)^. Until the end of August, Portugal recorded 58,243 confirmed cases and 1,824 deaths caused by the COVID-19; the incidence rate was 573.5 *per* 100,000 inhabitants^(8)^, which was pointed out as a case of relative success in comparison to the European Union and at a worldwide level.

However, the successful strategy was not repeated when a second epidemic wave emerged in which these figures grew vertiginously, with 3,549 new deaths and 282,044 new cases recorded up to December 2020^(9)^. The country’s initial success in fighting the pandemic’s first wave was partially attributed to the population’s ability to adhere to the government’s mitigation measures. However, studies^(10-11)^ show that some population groups may have failed in adhering to these measures, such as the population of men who have sex with men (MSM).

Because it is a population historically marginalized and its sexual practices are socially rejected, its activities are restricted to invisible/hidden places. For this reason, the social support provided by peers might be even more critical and necessary. It would be why this population does not fully adhere to social isolation measures and maintain risky sexual behaviors. Such behaviors significantly increase the likelihood of being infected by the SARS-CoV-2 and should be better clarified^(9)^.

These behaviors include chemical sex or sex under the influence of drugs^(10-12)^, commonly called “*chemsex”,* an association between the words chemical and sex. This practice is a common phenomenon in the community of men who have sex with men^(13)^ and has received particular attention in the field of Public Health due to its growing prevalence among MSM in both developed^(13)^ and developing countries^(14)^.

The type of drugs used in *chemsex* varies according to each country’s specificities and context; however, the literature^(15-16)^ reports that the most prevalent illegal drugs are: methamphetamine, poppers, gamma-butyrolactone (GHB/GBL), crystal methamphetamine, cocaine, erectile dysfunction drugs, ketamine, ecstasy, amphetamine, mephedrone and *cannabis*/marijuana. Drugs can be consumed in isolation or combined, which increases the potential risk of overdose and chemical dependency among users^(17)^.

Depending on the substance used, the perception of risk and adoption of protective measures may be impaired^(18)^ and as suggested by a multi-center study^(11)^, investigating the practice of *chemsex* among MSM during social distancing can provide important information regarding the emergence and rapid growth of new cases of COVID-19 in Portugal, a subject that has not been explored thus far.

In this context, this study’s objective was to investigate the factors associated with the practice of sex under the influence of drugs (chemsex) among Portuguese men who have sex with men during the period of social distancing to prevent the COVID-19.

## Method

### Study design

This online survey is part of a macro-project called “40TENA”, derived from the cohort “In_PrEP Brazil/ Portugal”, and was conducted in Portugal and Brazil, led by the Portuguese Institute of Hygiene and Tropical Medicine (IHMT) in partnership with the University of São Paulo (USP).

### Study setting and period

The study was conducted in 15 out of the 18 districts in Portugal in May, 2020 at the peak of the COVID-19 pandemic, when Portugal was under restrictive sanitary measures (social distancing and isolation and mandatory quarantine) advising the population to shelter at home and avoid interactions with people outside their household as much as possible.

### Sampling and participants

A standard sampling calculation for proportions was performed using G Power (version 3.1.9.7)^(19)^, considering the population of Portuguese men over 18 years of age with an assumed prevalence of 50% to maximize the sample, also considering it is a new phenomenon and there were no data concerning its prevalence)^(20)^ with a tolerable standard error of 3% and a confidence level of 95%, the final sample was 1,301 MSM.

### Data collection

The participants were recruited using snowball sampling adapted to the virtual environment^(11-12,21)^. In this method, the participants are responsible for recruiting other individuals with similar characteristics using social media. Fifteen MSM with different social and economic characteristics (commonly related to selection bias) were initially selected considering: country’s region, race (Caucasian and non-Caucasian), age (young, adult or elderly) and educational level. These participants, called seeds, received a link that granted access to the survey after they provided their consent to participate. They were asked to invite other MSM from their social network to participate. Two of the most popular worldwide geo-based dating apps (Grindr and Hornet) were used to identify the initial seeds^(21)^. The individuals were approached via online chat and data were collected by adapting the Time Location Sampling (TLS) technique to the virtual environment. The researchers purposely modified their location in the application for the regions selected to access a given area’s users, following the methods previously described^(22-24)^.

The researchers also promoted the survey on Facebook^®^, directing it to the MSM population aged from 18 to 60 years old (a Facebook-imposed age restriction), using a fixed post on the official page of the survey (https:// www.facebook.com/taafimdeque/), accompanied by an electronic link that granted access to a Free and Informed Consent Form (FICF) and the survey’s questionnaire. This procedure is essential to ensure that MSM living outside large cities or metropolitan areas are included^(23)^.

Only individuals who identified themselves as men (cisgender or transgender), 18+ years old and residing in Portugal were included; non-Portuguese speakers or tourists were excluded.

The online form was hosted by a specific data collection site that enabled only one response *per* IP (internet protocol), i.e., only one response *per* electronic device, to avoid selection bias. The form was content validated^(25)^ by three expert/specialist in the topic and was divided into four sections with 46 questions, most were multiple-choice questions, and some were mandatory; without answering those questions, the participant could not proceed with the questionnaire. The questions addressed social and demographic information (age, education, gender identity, housing and type of relationships, according to prior studies)^(10-11,26-27)^, mental health (self-perception of stress and strategies to cope with the pandemic), behaviors adopted to protect against the pandemic (social distancing, protective measures against the COVID-19 and level of adherence) and sexual behavior during the social distancing, isolation or quarantine (casual sex, adoption of protective measures against the COVID-19 and sexually transmissible infections [STIs], and type and number of sexual partners) and in the period immediately before the emergence of the pandemic (strategies to find partners, type and number of partners and preventive measures against STIs).

In this study, the participants were asked whether they had consumed drugs immediately before and/or during sexual intercourse since restrictive measures had been imposed in Portugal. Those who answered “yes” were asked to check the drugs consumed in a multiplechoice list. Considering there is not a universally accepted definition of what drugs compose the “chemsex phenomenon”^(28-29)^, in this study, both legal and illegal drugs that alter an individual’s perception and lead to the neglect of protective measures against SARSCoV-2 were included: alcohol; opioids (e.g., heroin, codeine and other synthetic substances); cannabinoids (marijuana, *hashish*, synthetic cannabinoids, spice); sedatives or hypnotics (barbiturates, benzodiazepines); cocaine; stimulants (e.g., amphetamines); hallucinogens (LSD; ecstasy); sex-performance-enhancing drugs (poppers) and others. To facilitate the identification and discrimination of drugs, some drugs were identified by other nomenclatures commonly used in Portugal. We also included an open category, “other drugs,” in which the participants could specify the drug used if it was not already listed.

Social distancing measures (also called, by the participants as social isolation and/or quarantine) were defined as avoiding personal contact for non-essential activities with people outside one’s shelter^(10)^ and an assessment and measurement of compliance to measures followed the literature’s guidelines^(10,26,30-31)^.

### Data analysis and treatment

Data were analyzed using the Statistical Package for the Social Sciences - SPSS v.26. Descriptive statistics such as absolute and relative frequencies were considered. Bivariate and multivariate analyses were performed with the chemsex practice being the dependent variable and sociodemographic variables, sexual behavior and strategies to prevent COVID-19 as the independent variables. Tolerance coefficients and VIF (Variance Inflation Factor) were verified to assess multicollinearity and proceed with the multivariate analysis. Odds Ratio (OR) and adjusted Odds Ratio (aOR) were used to measure the intensity of association between chemsex and associated factors considering their respective 95% confidence intervals. The model was developed using the stepwise method and the best goodness of fit and performance, according to the Hosmer and Lemeshow test, were considered.

### Ethical aspects

The Institutional Review Board at the Instituto de Higiene e Medicina Tropical (IHMT) at the Universidade Nova de Lisboa approved the study (protocol 1219/2020).

## Results

**Table 1:** Social, demographic and sexual health characterization of men who have sex with men (n: 1,301). Portugal, 2020

Interest variable	Chemsex^*^					%	p^†^
	Yes		No		Total		
	n	%	n	%	N		
**Sociodemographic characteristics**							
**Age (years old)**							0,001
<35	207	21,3	763	78,7	970	74,5	
≥ 35	56	16,9	275	83,1	331	25,4	
**Gender identity**							0,096
Cisgender man	251	19,6	1030	80,4	1281	98,4	
Transgender man	12	60	8	40	20	1,6	
**Education (complete years)**							0,001
≤9	52	77,5	311	85,7	363	27,9	
>9	211	22,5	727	14,3	938	72,1	
**Lives in the metropolitan region**							
Yes	252	95,8	955	92	1207	92,8	
No	11	4,2	83	8	94	7,2	0,033
**Current relationship**							0,001
Stable	28	10,6	173	16,7	201	15,5	
Open/polyamory	7	2,7	82	7,9	89	6,8	
Single	228	867	783	75,4	1011	77,7	
**Live with sexual partner**	18	6,8	149	14,4	167	18,6	0,001
**Use dating apps to seek partners**	214	81,4	774	74,6	988	75,9	0,021
**Sexual health**							
**Tested for HIV** ^‡^ **in the last 12 months**	106	40,3	559	39,3	665	57,1	0,239
**HIV**^‡^ diagnosis							
Unknown	12	4,6	124	11,9	136	10,4	
HIV^‡^ -	206	78,3	728	70,1	934	71,8	0,002
HIV^‡^ +	45	17,1	186	17,9	231	17,8	
**Use PrEP** ^§^ **/ Truvada** ^‖^	104	29,2	252	70,8	356	30,6	0,001

* Chemsex = Sex under the influence of drugs; ^†^p = Statistical significance obtained through a Chi-square test; ^‡^HIV = Human Immunodeficiency Virus; ^§^PrEP = Pre-exposure Prophylaxis; ^||^Truvada = brand name for the combination of tenofovir disoproxil fumarate (TDF – 300 mg) and emtricitabine (FTC – 200 mg)

A total of 1,301 MSM took part in the study. The participants were aged 30.5 years old on average (standard deviation=9.2; minimum 18 and maximum 66 years old) with a median of 1 sexual partner (minimum: 0 – maximum: 32) during the period of social isolation. Chemsex was reported by 20.2% (n=263) of the participants; all of whom (n=263) reported casual sex, and slightly less than half (44.9%; n=118) did not used condoms during sexual intercourse. Most sex partners practicing chemsex were found through dating apps (81.4%; n=214). Approximately 30% (n=356) of the participants used pre-exposure prophylaxis (PrEP/Truvada) against Human Immunodeficiency Vírus (HIV), as shown in Table 1.

Behaviors adopted during social distancing measures imposed to prevent the spread of COVID-19 seem to be associated with chemsex. The following variables stand out: the number of partners (p<0.001), HIV status (p=0.002), unprotected sex (p<0.001), group sex (p<0.001) and the adoption of preventive measures to avoid COVID-19, such as: to avoid kissing during sexual intercourse (p=0.003), hand washing during encounters (p=0.003), the use of PrEP (p<0.001) and COVID-19 testing (p<0.001), as shown in Table 2.

**Table 2:** Descriptive characteristics of sexual practice among men who have sex with men (n: 1,301) during the COVID-19 pandemic, considering chemsex*. Portugal, 2020

Interest variables	Chemsex^*^						p-value^†^
	Yes		No		Total		
	n	%	n	%	N	%	
**Social distancing period**							
**Duration of social isolation/distancing (days)**							0,060
Less than 29	44	16,7	114	11	158	12,1	
Between 30 and 45	116	44,1	502	48,4	618	47,5	
More than 45	88	33,5	374	36	462	35,5	
Did not adhere to social isolation	15	5,7	48	4,6	63	4,9	
**How many partners did you have since social distancing measures were imposed?**							0,001
None	0	0	466	44,9	466	35,8	
1 partner	20	7,6	397	38,2	417	32,0	
More than 2	243	92,4	175	16,9	418	32,2	
**During the social distancing you:**							
**Had sex with penetration without condoms**	145	55,1	121	11,7	266	20,5	0,001
**Paid for sexual intercourse**	16	6,1	35	3,4	51	3,9	0,043
**Group sex (sex with 2 or more people simultaneously)**	156	59,3	35	3,4	191	14,7	0,001
**Protective measures adopted in casual sex to prevent COVID-19**							
**Avoided kissing during sexual intercourse**	147	55,9	195	18,8	342	48,7	0,003
**Sanitized the place where you had sex**	143	54,4	198	19,1	344	48,8	0,017
**Washed hands with water and soap**	148	56,3	197	19	345	49,9	0,003
**Verified whether the sexual partner had signs and symptoms of COVID-19**	158	60,1	457	35,1	615	63,7	0,031
**Used PrEP** ^‡^ **/ Truvada**	112	42,6	64	6,2	176	25,1	0,001
**COVID-19 testing**							
Yes	83	31,6	167	16,1	250	19,2	
No	180	68,4	871	83,9	1051	80,8	0,001
**Diagnosed with COVID-19**							
Yes	41	15,6	58	5,6	99	39,6	0,026
No	42	16	109	10,5	151	60,4	

* Chemsex = Sex under the influence of drugs; ^†^p = Statistical significance obtained through a Chi-square test; ^‡^HIV = Human immunodeficiency Virus; ^§^PrEP = Pre-exposure Prophylaxis

Bivariate and multivariate logistic regression was used and the following factors appeared associated with chemsex during the COVID-19 pandemic among Portuguese MSM. Those who practiced group sex during quarantine measures were 28 times more likely to engage in chemsex, while those who tested for COVID-19 were 1.9 times more likely to engage in chemsex*.* Those MSM who had sex without protection were 7.1 times more likely to engage in chemsex, while those who reported PrEP as a way to prevent COVID-19 were 4.2 times more likely to engage in chemsex (Table 3).

**Table 3:** Bivariate and multivariate analyses considering men who have sex with men (n: 1,301) reporting chemsex during the COVID-19 pandemic. Portugal, 2020

Variables	OR^*^	p-value^†^	95%CI^‡^	aOR^§^	p-value^†^	95%CI^‡^
**Sociodemographic characteristics**						
**Current relationship**						
Stable	1					
Polyamory/open	0,52	0,145	0,22 – 1,25			
Single	1,79	0,007	1,17 – 2,75			
**Live with sexual partner**						
Yes	1					
No	2,42	0,001	1,44 – 4,07			
**Live in the metropolitan area**						
No	1					
Yes	1,99	0,030	1,04 – 3,79			
**Use dating apps to seek partners**						
No	1					
Yes	1,49	0,022	1,06 – 2,09			
**Sexual health**						
**HIV**^‖^ Status						
Unknown	1					
HIV^‖^ -	2,92	0,001	1,58 – 5,39			
HIV^‖^ +	2,50	0,005	1,27 – 4,91			
**During the social distancing period Group sex (with 2 or more people simultaneously)**						
**Sexo en grupo (simultáneo con 2 o más personas)**						
No						
Yes	41,78	0,001	27,52 – 63,41	28,4	0,001	16,93 – 47,49
**Sex with penetration without condoms**						
No						
Yes	9,31	0,001	6,84 – 12,67	7,1	0,001	4,57 – 10,99
**Paid for sexual intercourse**						
No	1					
Yes	1,85	0,040	1,01 – 3,40			
**Protective measures against the COVID-19 adopted during casual sex**						
**Disinfected the place where you had sex**						
No	1					
Yes	1,45	0,018	1,06 – 1,97			
**Avoided kissing during sexual intercourse?**						
No	1					
Yes	1,58	0,003	1,16 – 2,15			
**Verified whether the sexual partner had signs and symptoms of COVID-19**						
No	1					
Yes	1,41	0,031	1,03 – 1,95			
**Used PrEP** ^¶^ **/ Truvada**						
No	1					
Yes	4,34	0,001	3,03 – 6,23	4,2	0,001	2,71 – 6,39
**COVID-19 Investigation**						
**COVID-19 testing**						
No	1					
Yes	2,40	0,001	1,76 – 3,27	1,9	0,012	1,15 – 3,10

* *OR = Odds Ratio; ^†^p = Statistical significance; ^‡^95%CI = 95% Confidence Interval; ^§^aOR = adjusted Odds Ratio; ^||^HIV = Human Immunodeficiency Virus; ^¶^PrEP = Pre-exposure prophylaxis. Hosmer and Lemeshow test (p=0.61)

## Discussion

The data from this study show a high frequency of chemsex (20.2%) in this population, considering the results reported by a research addressing this same population before the pandemic (9.2%)^(29)^. In this sample, one in every four MSM engaged in casual sex using at least one substance capable of changing cerebral functioning and altering an individual’s mental and psychological state. These results are generally of concern; however, they are even more critical considering that the chemsex practice increased^(10,29-30)^ precisely when the epidemiological curve of the first wave of COVID-19 in Portugal (April and May 2020) was ascending.

The large portion of MSM who reported chemsex during the social isolation period in the country may be related to the fact that data were collected only a few days after the first state of emergency was issued in Portugal, when some of the participants behaved the same as to the period before the pandemic. Throughout the prolonged period of social distancing, psychological disorders such as anxiety and depression^(32-33)^ became evident and casual sex with unknown partners, drug use and multiple partners may have been adopted as a way to “relax and escape from reality” even if for a short period and with risks.

Among the factors that increase the chances of MSM engaging in chemsex*,* goup sex (with 2 or more people simultaneously) stood out as conferring a 28 times greater chance than among those who did not report sex group. This is a classic relationship reported in the literature, in which studies^(34-35)^ report that group sex is usually associated with the use of drugs, regardless of the type of drug, though those that enhance sexual performance, such as mephedrone, methamphetamine and/or GHB/ GBL stand out.

Those practicing chemsex associated it with significant improvements in quality of life and sex performance because it decreases inhibition and increases sexual arousal and pleasure. The combination of drugs, long sex sessions, and multiple partners also leads to even more challenging sexual practices such as fisting and duple penetration^(36)^. This finding highlights the possibility of overlapping exposure and synergistic epidemic, considering that the use of drugs may hinder adherence to measures intended to prevent Sexually Transmitted Infections (STIs) while simultaneously, the reunion of people with different levels of exposure to SARS-CoV-2 in the same place for a high amount of time may increase the likelihood of being contaminated by the novel coronavirus^(36-37)^.

One finding that illustrates this situation is that the practice of anal sex without condoms increased 7.1 times the likelihood of an individual engaging in chemsex*.* This piece of information reinforces that MSM who practice chemsex might be more willing to engage in risky behavior. The use of illegal or legal drugs in a sexual context leads to decreased discernment among vulnerable populations such as MSM, considering they are more likely to acquire HIV and STIs, highlighting the risks they are exposed to^(29)^. In a context of the coronavirus pandemic, the likelihood of new cases emerging among the Portuguese population increase due to sexual behavior and a high risk of acquiring the COVID-19^(14)^.

This study’s results corroborate previous studies^(38-41)^, which report that the use of drugs in a sexual context considerably increases risky behaviors (unprotected sexual intercourse) due to decreased risk perception. Hence, MSM who practice *chemsex* are more likely to acquire HIV and other STIs, to practice group sex or sex with more than one partner in the same night, to decrease adherence to the antiretroviral treatment among HIV positive patients and to protective measures such as de PrEP, which was reported by approximately 30% of the MSM addressed in this study.

However, the sample of MSM addressed in this study adopted PrEP not only to prevent HIV. This group, mistakenly, adopted PrEP as a way to prevent SARSCoV-2 as well. These individuals’ chances to engage in chemsex increased 4.1 times, a result that was also found among Brazilian MSM^(11)^. The hypothesis^(10)^ is that, given the misunderstanding reported by the mass media and social media, such as Facebook, concerning the prophylactic medications’ potential to fight SARS-CoV-2^(42)^, some MSM may have confused the PrEP strategy. That is, they assumed the HIV pre-exposure prophylaxis had a similar effect (prophylaxis) against the SARS-CoV-2, which may have motivated the maintenance of sexual practices during the pandemic.

These beliefs may also have been influenced by the dissemination of recent preliminary studies^(43-44)^ conducted in Brazil investigating the potential of Tenofovir, one of the antiretroviral drugs used in Truvada, to decrease the length of hospitalizations due to SARS-CoV-2. Such information enhances the ability of fake news to spread on social media. Since there is no evidence it prevents COVID-19, there is an increased risk of people using PrEP neglecting the effective protective measures^(45)^ recommended by sanitary agencies during the social isolation and being infected with SARS-CoV-2.

Another questionable measure, but which increased the likelihood of MSM engaging in *chemsex,* is based on the COVID-19 testing as those who reported testing for COVID-19 were, almost, 2 times more likely to engage in chemsex than those who did not.

We believe that the testing may lead to a false sense of security. A belief that individuals who tested positive for COVID-19 acquire immunity against the disease, hence would not be re-infected, may have led MSM to take more risks. However, this is a misleading belief, considering that it is unknown how immunological memory^(46-47)^ works with this new infection or what its efficacy is. Additionally, the literature has already reported re-infection by different strains^(48)^ of the same virus.

On the other hand, the participants who tested negative for COVID-19 may have been encouraged to have sex with unknown partners for believing that “they do not get infected”, especially when signs and symptoms of COVID-19 were absent. The literature corroborates this finding. A Brazilian study reports that MSM who tested recently were more prone to engage in casual sex. Among the measures adopted to manage the risk of acquiring SARS-Cov-2, verifying that partners did not present signs and symptoms of COVID-19 was determinant for maintaining sexual encounters^(12)^.

The findings of this study are unprecedented in the literature and, coupled with other similar^(10-12)^, raised questions regarding the actual role and impact of sexual behavior and the maintenance of certain risky behaviors (e.g., casual sex, group sex, with the use of drugs or without condoms) on the current COVID-19 pandemic. This panorama shows that good mitigation strategies (social distancing/isolation or quarantine) pervade messages addressing sexual issues and vulnerable populations. Disseminating information regarding the practice and use of less harmful drugs with a decreased number of people or at least decreased sexual partner turnover and, simultaneously, encouraging preventive measures against STIs and COVID-19 may be effective.

This study presents some limitation. The first referred to the impossibility of establishing a causal relationship between the practice of chemsex and SARS-COV-2 infection or stating that the maintenance of isolation measures increased the occurrence of risky sexual behavior for STIs or COVID-19. The online collection of data was based on self-reported data, addressing an accidental sample and even though mechanisms were used to diversify the sample, a lack of a more robust sampling calculation (e.g., cluster sampling) restricts the inference of results in addition to the possible restriction among participants who were more familiar, and with greater access, to virtual tools. These findings should be interpreted with caution, primarily because behaviors and circumstances related to the COVID-19 may change suddenly.

## Conclusion

The occurrence of chemsex among MSM during the COVID-19 pandemic in Portugal was very high, showing an increase in its prevalence. It indicates that the worldwide health calamity did not sensitize Portuguese MSM to adhere to restrictive and social distancing measures. Aspects such as age, education, gender identity, type of relationship, the adoption of dating apps, HIV and COVID-19 testing, duration of isolation, number of sexual partners, sexual behavior during the quarantine and protective measures against the COVID-19 were significantly associated with chemsex among MSM in Portugal. These results reveal that it is impossible to ignore the role of sexual and loving relationships and activities on the adherence to social distancing measures and the magnitude of the pandemic itself, especially among socially marginalized groups such as MSM.

We suggest that messages recommending preventive measures against the COVID-19 explicitly target sexual behavior and its consequences for the aggravation of the current pandemic context be part of governmental actions.
